# High coral reef connectivity across the Indian Ocean is revealed 6–7 Ma ago by a turbid-water scleractinian assemblage from Tanzania (Eastern Africa)

**DOI:** 10.1007/s00338-019-01830-8

**Published:** 2019-06-24

**Authors:** Markus Reuter, Francesca R. Bosellini, Ann F. Budd, Stjepan Ćorić, Werner E. Piller, Mathias Harzhauser

**Affiliations:** 1 Institute of Geophysics and Geology, University of Leipzig, Talstraße 35, 04103 Leipzig, Germany; 2 Department of Chemical and Geological Sciences, University of Modena and Reggio Emilia, Via Campi 103, 41125 Modena, Italy; 3 Department of Earth and Environmental Sciences, University of Iowa, 115 Trowbridge Hall, Iowa City, 1A 52242 USA; 4 Geological Survey of Austria, Neulinggasse 38, 1030 Vienna, Austria; 5 Institute of Earth Sciences, University of Graz, NAWI Graz Geocenter, Heinrichstraße 26, 8010 Graz, Austria; 6 Department of Geology and Palaeontology, Natural History Museum Vienna, Burgring 7, 1010 Vienna, Austria

**Keywords:** Turbid reef, Coral palaeobiogeography, Ocean currents, Rovuma Delta, Indo-West Pacific, Miocene

## Abstract

The present centre of coral diversity in the Western Indian Ocean is defined by the northern Mozambique Channel with an extension northward to Mafia Island in Tanzania (Eastern Africa). The geological and evolutionary history of this hotspot of marine biodiversity remains so far completely obscure, because Cenozoic fossil reef communities of this area are not well known. This study presents a new fossil scleractinian fauna from the Mikindani Formation in southern Tanzania. It comprises 16 symbiotic coral taxa of which nine could be identified to the species and five to the genus level. Coral habitat consisted of low-relief biostromes that developed in shallow water at the front of the Rovuma Delta under conditions of variable sediment input. The biostromes are dated to be Messinian based on associated calcareous nannoplankton and planktic foraminifers. The studied coral assemblage shows close affinities with the Recent Western Indian Ocean biogeographic province and Central Indo-West Pacific biogeographic region as well as with the Miocene of Indonesia. Faunistic relations with the Oligocene-early Miocene of Somalia and Iran do not exist. The patterns of species distribution document a major palaeobiogeographic change in the Indian Ocean that correlates with the onset of the Miocene Indian Ocean Equatorial Jet during the middle Miocene. The clear Indonesian affinity of the Messinian coral fauna from southern Tanzania implies that this westerly oceanic surface current provided high biogeographic connectivity across the Indian Ocean during the late Miocene. Today, the coastal waters of Indonesia are located in the Coral Triangle. Diversification of this global epicentre of marine biodiversity started in the early Miocene and it was established already during the middle Miocene. Our results indicate that the East African hotspot of coral biodiversity originated as an offshoot of the Coral Triangle in the middle to late Miocene.

**Electronic supplementary material:**

The online version of this article (10.1007/s00338-019-01830-8) contains supplementary material, which is available to authorized users.

## Introduction

The Indo-West Pacific is the largest biogeographic realm of the world’s oceans, comprising the tropical waters of the Indian Ocean (Western Indo-West Pacific biogeographic region), the western (Central Indo-West Pacific biogeographic region) and central Pacific Ocean (Eastern Indo-Pacific biogeographic region), and the seas connecting the two in the general area of the Malay Archipelago (Central Indo-West Pacific biogeographic region; Spalding et al. 2007). The latter region includes the most important centre of marine biodiversity (across diverse taxa) on Earth (Tittensor et al. 2010). This biodiversity hotspot is a vast network of coral reef in the waters surrounding the Philipines, Indonesia, Malaysia, Papua New Guinea, the Solomon Islands and Timor-Leste and has been named the Coral Triangle (Hoeksema 2007). 76% of the world’s coral species and 37% of the known reef fish species live there (Hoegh-Guldberg et al. 2009). A subordinate centre of coral reef biodiversity occurs in the northern Mozambique Channel (Eastern Africa; Reaka et al. 2008; Obura 2012; Veron et al. 2015; Förderer et al. 2018).

Over the Cenozoic, the emergence and movement of biodiversity hotspots on tropical coral reefs were primarily driven by plate tectonics that have substantially increased the area and physiological complexity of shallow-water habitats and brought together previously distinct biogeographic provinces (Wilson and Rosen 1998; Renema et al. 2008; Leprieur et al. 2016). The fossil evidence from the Coral Triangle suggests a period of rapid reef coral diversification during the early Miocene followed by a plateau of relatively high palaeobiodiversity (Johnson et al. 2015; Santodomingo et al. 2015a, 2016). The formation of the biodiversity hotspot in the early Miocene corresponds to a phase when new islands and shallow seas were extensively created by the collision of Australia with Pacific arcs and the southeast Asian margin (Renema et al. 2008). In contrast to the Coral Triangle, the Western Indian Ocean centre of coral reef biodiversity has no fossil record and, accordingly, the geological and evolutionary origins of this species richness are totally unknown.

Here, we report on a reef coral assemblage from the Mikindani Formation of Miocene–Pliocene age in southern coastal Tanzania and reconstruct the palaeoenvironment. The siliciclastic Mikindani Formation represents the Rovuma Delta, one of the largest Cenozoic delta systems on Africa’s east coast (Key et al. 2008). In terms of recent coral biogeography, the Rovuma Delta belongs to the Central (or “Core”) ecoregion of the Western Indian Ocean biogeographic province, which hosts the maximum of coral richness in the Western Indo-West Pacific (Obura 2012; Veron et al. 2015). Calcareous nannoplankton and planktic foraminifers provide the biostratigraphic framework for a comparison of the new East African coral fauna with known fossil faunas from different regions of the Indo-West Pacific and the Mediterranean. From this comparison, we expect to better understand the history of coral reef biodiversity in the Western Indian Ocean.

### Geological background and setting

**Fig. 1:**
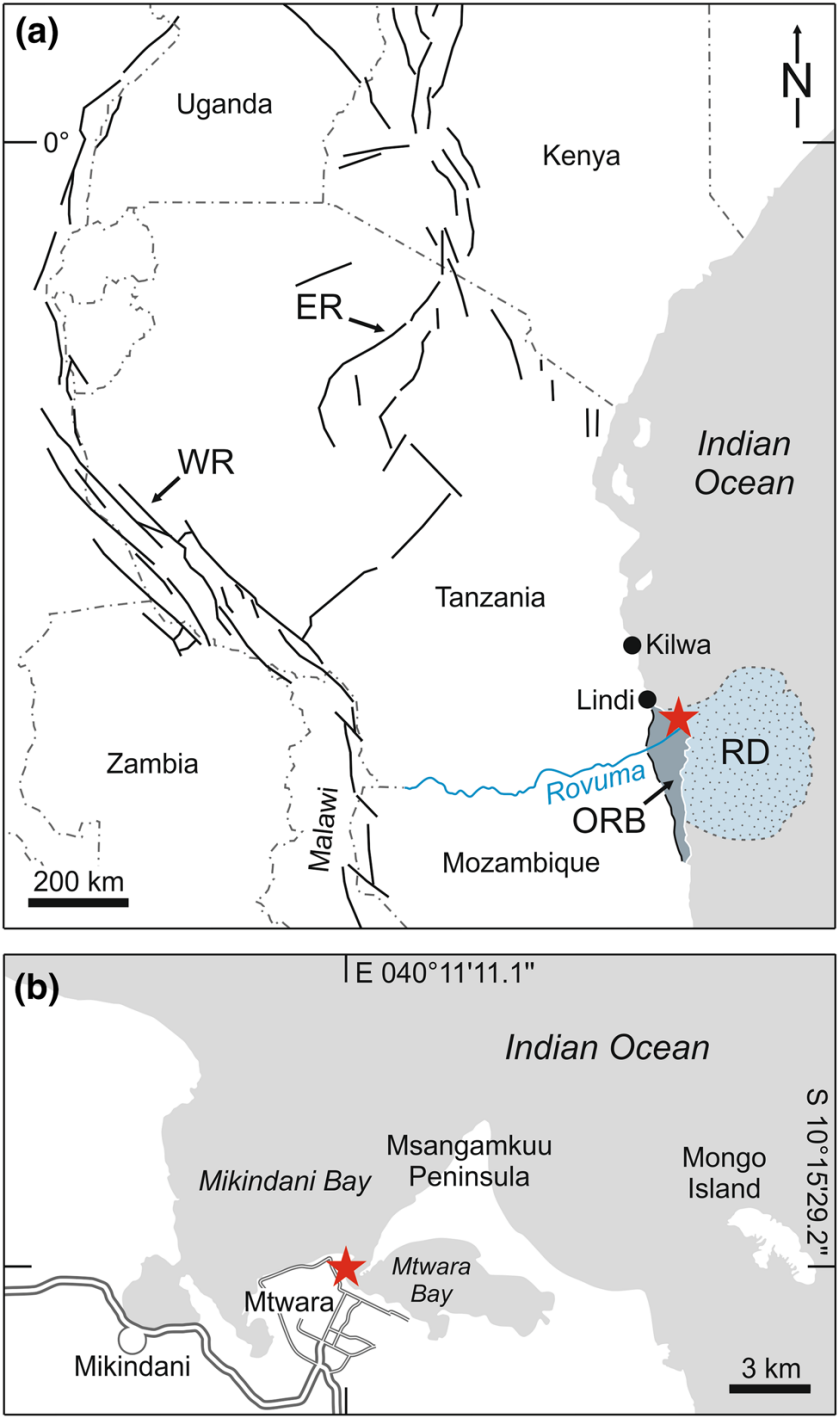
Study site. **a** Overview map showing the distribution of Cenozoic rifts in Tanzania and adjacent regions (ER = Eastern Rift, WR = Western Rift), the course of the Rovuma River, the position of the study site (red asterisk) in the onshore Rovuma Basin (ORB/dark blue) and the extent of the Rovuma Delta (RD/light blue) off East Africa. **b** Detail map of the Mtwara area with location of the cliff outcrop (red asterisk)

This study was carried out in the northern Rovuma (or Ruvuma) Basin at a sea cliff ca 500 m north of Mtwara fish market in Mtwara Bay (southern coastal Tanzania; S10°15′29.2″, E040°11′11.1″; Fig. 1b). The NNW–SSE trending Rovuma Basin is about 400 km long and 160 km wide and extends onshore in southern Tanzania and northern Mozambique on the East African passive continental margin (Fig. 1a); offshore it continues towards the Davie Fracture Zone (Salman and Abdula 1995; Smelror et al. 2006, 2008; Key et al. 2008; Mahajane 2014). The basin is centred on the Rovuma Delta (Fig. 1a) in the area between the coastal town Mtwara (southern Tanzania) and Cape Delgado (northern Mozambique). Basin history was directly linked to the progressive break-up of southern Gondwana, and the sedimentary succession of the basin can be divided into five tectono-stratigraphic mega-sequences reflecting different stages of break-up. The final stage is marked by the progradation of an easterly thickening wedge of deltaic sediments offshore the Rovuma River from the Oligocene onwards (Salman and Abdula 1995; Smelror et al. 2006; Key et al. 2008). Its formation was probably initiated by regional uplift of eastern Africa, linked to a doming during the Oligocene (Key et al. 2008) or at an earlier stage during the Lutetian (Roberts et al. 2012), preceding the formation of the eastern branch of the East African Rift System (Fig. 1a). It is claimed that this uplift modified continental drainage patterns and directions for major large river systems including the Nile, Congo and Zambezi (Roberts et al. 2012) and likely also the Rovuma (Mahajane and Franke 2014). The Miocene marks a period of active extension along the coast of southern Tanzania that created accommodation space for deltaic sediment accumulation linked to the southwards propagation of the eastern rift branch (Nicholas et al. 2007; Fig. 1a). Additionally, rotational block faulting caused a complex basin topography at this time, as recorded by spatially heterogeneous facies patterns in the present-day coastal zone between the towns Kilwa and Lindi (Nicholas et al. 2007). In this region, which was unaffected by deltaic sedimentation (Fig. 1a), thick pelagic clay deposits formed on the top of rotated hangingwall blocks, whereas carbonate platforms developed in shallower settings on the uplifted crest of footwall blocks. Isolated coral patch reefs were suggested to have occupied topographic highs further inshore towards the palaeocoastline (Nicholas et al. 2007). Such a shallow-marine palaeoenvironment with corals is represented by a gastropod fauna of Aquitanian age that was discovered in an isolated block of limestone in the so-called “Geobreccia” of Ras Tipuli, about 5 km north of Lindi town (Harzhauser 2009). Differently to this locality, the fossil site at Mtwara is situated in the northern part of the Miocene Rovuma Delta (Fig. 1a).

## Materials and methods

The primary sedimentological and palaeontological dataset consists of semi-quantitative information gathered through field observations. A 10.5-m-thick stratigraphic section (MT-07) was measured bed by bed. Four samples of muddy matrix between corals were taken from beds 1, 2, 3 and 5 and processed for their foraminiferal content using standard wet sieving procedures with meshes > 0.8 mm, > 315 µm and > 125 µm. Foraminifers were identified by F. Rögl (Natural History Museum Vienna, Austria). Smear slides were prepared from the same samples for calcareous nannoplankton analyses using the standard procedures described by Perch-Nielsen (1985) and examined under a light microscope (parallel and crossed polarizers) with 1000× magnification. A fauna of 45 fossil coral specimens was collected from the scree at the foot of the cliff for precise taxonomic identification. The samples were chosen to cover a broad range of coral taxa having a good preservation of external surfaces, as most of the corals appear deeply recrystallized or are preserved as moulds. The fossils described in this study are stored in the collection of the Geological-Palaeontological Department of the Natural History Museum Vienna (Austria).

## Results

**Fig. 2:**
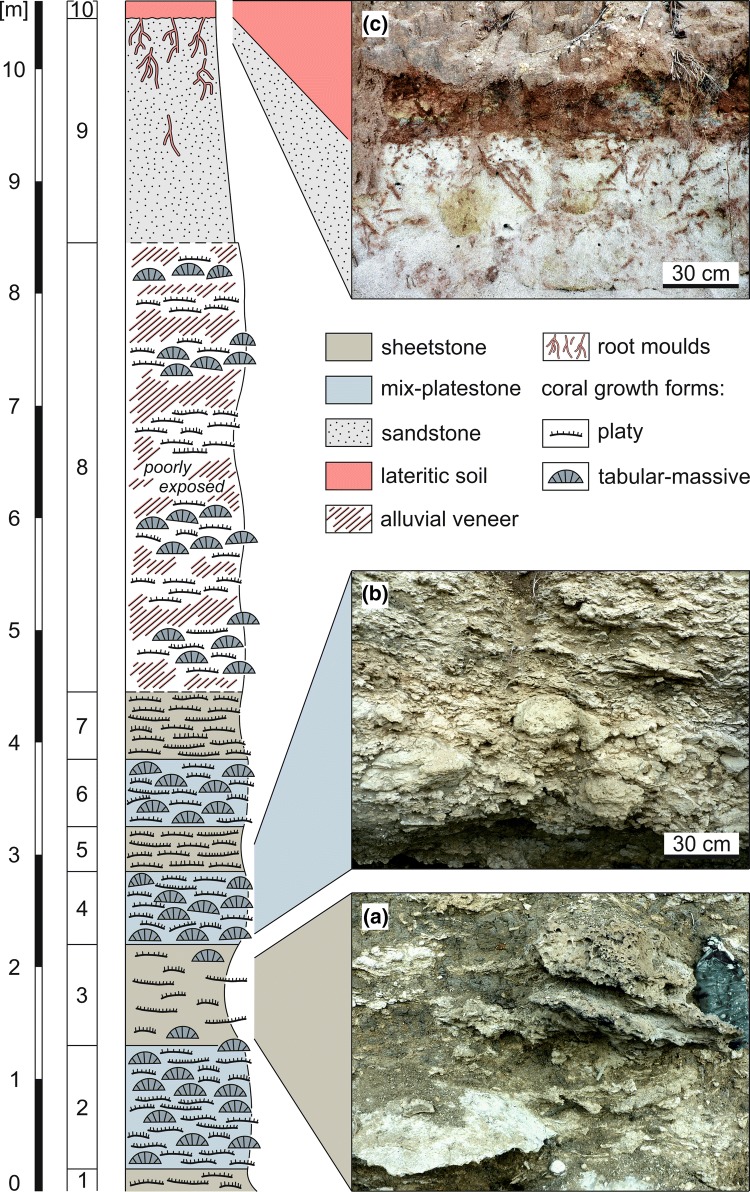
Mtwara cliff section, lithological log and main facies types. **a** Sheetstone facies. **b** Mix-platestone facies. **c** Lateritic soil capping at the top of the section. The contact to the underlying “Mikindani Beds” is sharp with soil-filled rootlets protruding into the quartz sand

**Fig. 3:**
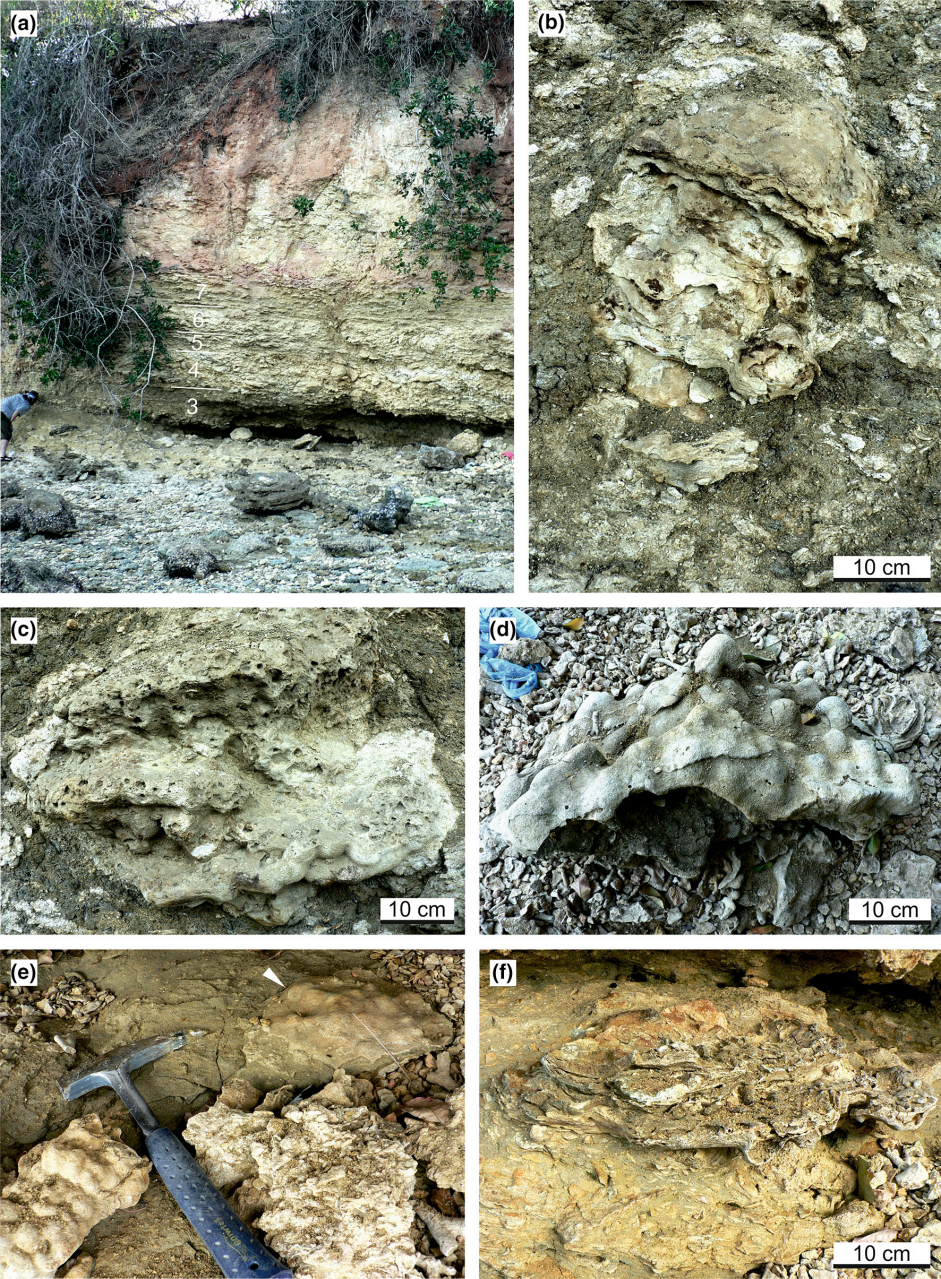
Architecture of the fossil coral bioconstruction in Mtwara Bay and coral growth features related to high sedimentation. **a** Stack of near-horizontal biostromes alternatingly dominated by platy and tabular-massive corals; the numbers refer to bed numbers in Fig. 2. **b** Massive *Porites* colony grown in columnar shape to keep pace with sedimentation. **b**, **c** Massive corals subjected to sedimentation pulses typically show ragged margins that resulted from partial mortality followed by growth of the surviving part of the colony; both corals in situ, bed 3. **d** Tabular-massive coral colony with ragged margins and protruding knobs preventing sediment accumulation; scree at the cliff base. **e** Convex knobs on the surface of a thin platy coral (white arrow head); in situ, bed 1. **f** Coral of highly irregular, laminar-interconnected shape that formed when sediment became lodged on concave areas of a platy colony; in situ, bed 3

The cliff outcrop at Mtwara exposes a coral bioconstruction of at least 8.5 m thickness (base is not exposed) over a distance of ca 25 m (Fig. 2). Corals in this bioconstruction are mostly in situ but do not form rigid frameworks. A total of sixteen taxa of symbiotic corals belonging to twelve genera have been identified from the outcrop (Table 1). Internally, the bioconstruction exhibits an indistinctive several decimetre- to metre-scale sub-horizontal bedding due to gradually changing growth fabrics and associated changes in the siliciclastic content (Figs. 2, 3a). Two principal facies are encountered based on the prevailing coral shapes and the type of matrix. The subdivisons are (1) siliciclastic (up to 80% mud-size siliciclastic material) sheetstone facies (sensu Insalaco 1998) dominated by platy colonies (beds 1, 3, 5, 7; Fig. 2a), and (2) carbonate (< 30% mud-size siliciclastic material) mix-platestone facies (sensu Insalaco 1998) including a variety of growth forms (platy, tabular-massive, branching and solitary; beds 2, 4, 6; Fig. 2b). Tabular-massive corals can reach a size of up to 50 cm across. Common growth features of the tabular-massive colonies are ragged margins (Fig. 3b, c). Corals with large internal sediment inclusions and interconnected platy to laminar growth (Fig. 3f) as well as flat colonies with protruding knobs (Fig. 3d, e) are also common. The lower part of the outcrop (beds 1–7) shows a regular, cyclic alternation of sheetstones and mix-platestones (Figs. 2, 3a). The upper part of the bioconstruction also contains sheetstone and mix-platestone facies but is not continuously exposed due to soil washed over the steep cliff edge (Figs. 2, 3a). The sediment between the corals contains cypraeid gastropods, pectinid bivalves, cidaroid echinoids, bryozoans, ostracods, benthic (*Operculina* sp., *Amphistegina* sp., *Heterolepa* sp.) and planktic foraminifers (*Globigerina bulloides*, *Gg*. *falconensis*, *Globigerinoides bisphericus*, *Gs*. *conglobatus*, *Gs*. *elongatus*, *Gs*. *extremus*, *Gs*. *obliquus*, *Gs*. *ruber*, *Gs*. *sacculifer*, *Gs*. cf. *tenellus*, *Gs*. *trilobus*, *Globigerinella* cf. *praesiphonifera*, *Globoquadrina* cf. *altispira*, *Gq*. *dehiscens*, *Globorotalia menardii*, *Gr*. *merotumida*, *Orbulina universa*, *Sphaeroidinellopsis seminulina*). Calcareous nannoplankton is represented by *Amaurolithus primus*, *Discoaster brouweri*, *D. quinqueramus*, *D. surculus*, *D. variabilis*, *Nicklithus amplificus* and *Reticulofenestra minuta, R. rotaria*, *Sphenolithus abies* and *S. moriformis*.

**Table 1:** Composition of the sampled coral assemblage and stratigraphic and geographic ranges of the identified taxa

Taxon	Number of collected specimens	Growth form	Stratigraphic range	Oligocene–Miocene fossil record				Recent occurrence
				Mediterranean	Tethyan Seaway	Western Indo-West Pacific	Central Indo-West Pacific	
*Acropora*	4	Branching	Eocene – Recent	N Italy (Ru, Ch)^1^, N Slovenia (Ru)^1^, Mesohellenic Basin (Ch)^2^, Gulf of Suez (Bur)^3^	Central Iranian Basins (Ch)^13^	Makran (Aq, Bur)^5^	Indonesia (Ru, Ch, Bur – Me)^9,11^	Indian Ocean, Pacific Ocean, Caribbean Sea
*Cycloseris*	1	Solitary	?Cretaceous – Recent			Sindh (Bur)^6^, Kenya (early Mio)^15^	Indonesia (Bur – Me)^9^	Indo-West Pacific, Eastern Pacific
*Fungia*	5	Solitary	Miocene – Recent				Indonesia (Lan – Me)^9^	Indo-West Pacific
Fungiidae indet.	4	Solitary (fragments)						Indian Ocean, Pacific Ocean
*Goniastrea edwardsi*	1	Tabular-massive	Miocene – Recent				Indonesia (Mio)^7^	Indo-West Pacific
*Goniopora planulata*	4	Tabular-massive	Miocene – Recent				Indonesia (Lan – Ser)^8,10^	Indian Ocean, Indonesian/Philippines Archipelago
*Hydnophyllia costata*	1	Fungiform	Oligocene – Miocene	N Italy (Ru, Ch)^4^				
*Lithophyllon*	1	Encrusting polystomatous	?Oligocene – Recent				Indonesia (Bur – Ser)^9^, Marion Platform (middle – late Mio)^12^	Central Indo-West Pacific
*Oulophyllia crispa*	1	Tabular-massive	Miocene – Recent				Indonesia (Ser – Tor)^10^	Indo-West Pacific
*Pachyseris affinis*	4	Platy	Miocene			Makran (Aq)^5^, Sindh (Bur)^6^	Indonesia (Mio)^7,10^	
*Pachyseris speciosa*	3	Platy (unifacial laminae)	Miocene – Recent				Indonesia (Aq – Ser, Me)^10^	Indo-West Pacific
*Platygyra concentrica*	1	Platy	Eocene – Miocene				Indonesia (Mio)^10^	
*Platygyra daedalea*	1	Platy	Miocene – Recent			Makran (Bur)^5^	Indonesia (Ser – Me)^10^	Indo-West Pacific
*Porites*	2	Massive, tabular-massive	Eocene – Recent		Central Iranian Basins (Ch – Bur)^13^	Somalia (Ch – Bur)^14^, Makran (Aq, Bur)^5^, Sindh (Bur)^6^	Indonesia (Oligo, Bur – Me)^9^	circumglobal
	1	Platy						
	9	Branching (fragments)						
*Turbinaria mesenterina*	1	Platy	Miocene – Recent					Indo-West Pacific
Undetermined	1							

Fossil occurrences from ^1^Wallace and Bosellini (2014), ^2^Wielandt-Schuster et al. (2004), ^3^Schuster (2002a), ^4^ Budd and Bosellini (2016), ^5^McCall et al. (1994), ^6^Duncan (1880), ^7^Santodomingo (2014), ^8^Santodomingo et al. (2015b), ^9^Santodomingo et al. (2016), ^10^Johnson et al. (2015), ^11^Santodomingo et al. (2015b), ^12^Conesa et al (2005), ^13^Schuster and Wielandt (1999), ^14^Bosellini et al. (1987), and ^15^Gregory (1930); the herein presented locality is not included. The Recent geographic distributions are based on Veron (2000); stratigraphic ranges according to the Paleobiology Database (https://www.paleobiodb.org, Accessed 10 April 2019) and Veron (2000) supplemented by this study (*H. costata*, *T. mesenterina*)

A coarse, unconsolidated, pale grey quartz sand (2-m thick) composed of angular grains cover the coral bioconstruction (bed 9; Fig. 2c). It comprises moulds of plant roots, which become increasingly abundant towards the top of the massive deposit. These are filled with red, coarse-grained clayey sand that is overlying bed 9 (bed 10; Fig. 2c). Pedogenic carbonate nodules (calcrete) are common in bed 10.

## Discussion

### Stratigraphy

The presence of *N. amplificus* allows a correlation of the coral-bearing deposits to the Calcareous Nannofossil Miocene Biozone (CNM) 18 of Backman et al. (2012). This zone is defined by the total range of *N. amplificus* covering the time interval between 6.82 and 5.98 Ma in the middle part of the Messinian. According to Backman et al. (2012), CNM18 corresponds to the upper part of NN11 (Martini 1971) and the middle part of CN9b (Okada and Bukry 1980).

Although not as precise as calcareous nannoplankton, the associated planktic foraminifers give a stratigraphic range for the Mtwara bioconstruction between late Tortonian and early Zanclean. *Globorotalia merotumida* occurs from M13a to PL1, *S. seminulina* has its highest occurrence at the top of Zone PL3 and *Globigerinoides extremus* ranges from M13a to PL6 (biozones after Wade et al. 2011). *Sphaeroidinella*, which appears first close to the Miocene/Pliocene boundary (Wade et al. 2011), is lacking in Mtwara section.

Coarse, unconsolidated quartz sands and grits, such as those covering the Mtwara bioconstruction (bed 9; Fig. 2), are widespread unconformably overlying Eocene and Miocene strata in the coastal zone of Tanzania, including the Mtwara region, and have been informally referred to the “Mikindani Beds” of Pliocene or younger age (Kent et al. 1971; Schlüter 1997; Nicholas et al. 2007). The type locality is the coastal town Mikindani (Bornhardt 1900) close to Mtwara (Fig. 1b). The “Mikandani Beds” representing a phase of significant uplift and erosion in southern Tanzania after the late Miocene due to doming and tilting which occurred across the Tanzania craton immediately before the western branch of the East African Rift System (Fig. 1a) was initiated. As a consequence, the coastal zone was effectively blanketed by fluvial and shallow-marine sands and grits of the “Mikindani Beds” (Nicholas et al. 2007). These sediments have been subsequently altered to lateritic soil in many places (Nicholas et al. 2007), including the study locality (bed 10; Fig. 2c). In a revision of the lithostratigraphy of the onshore Rovuma Basin in northern Mozambique, Key et al. (2008) formalized the Mikindani Formation so as to encompass variegated shallow marine or estuarine sands and sandstones that post-date the Oligocene Quissanga Formation and to be genetically linked to the Rovuma Delta Complex.

### Coral environment

**Fig. 4:**
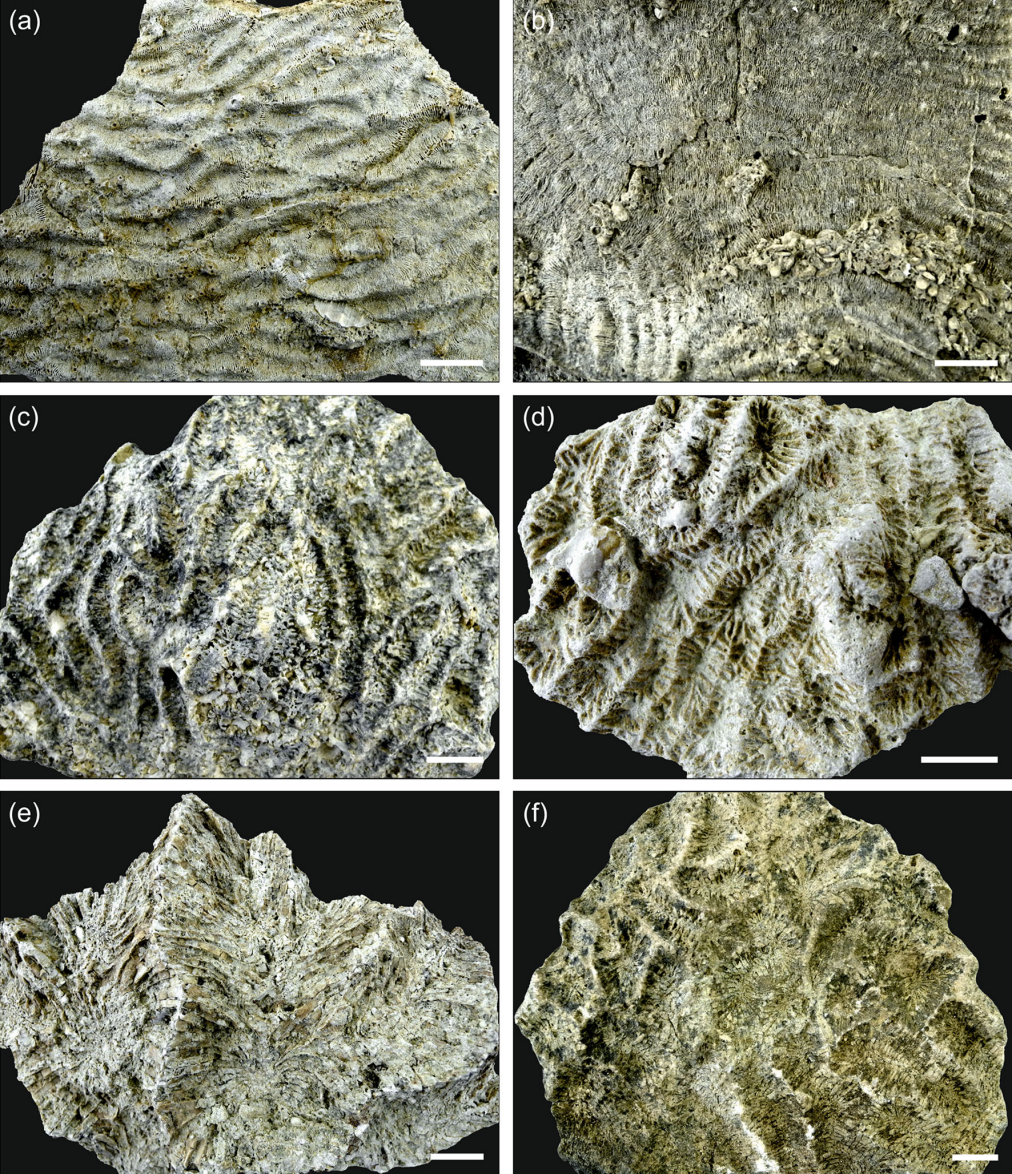
Representative corals from the studied assemblage. **a***Pachyseris affinis*. **b***Pachyseris speciosa*. **c***Platygyra concentrica*. **d***Platygyra daedalea*. **e***Hydnophyllia costata*. **f***Oulophyllia crispa*. The scale bar is always 1 cm

**Fig. 5:**
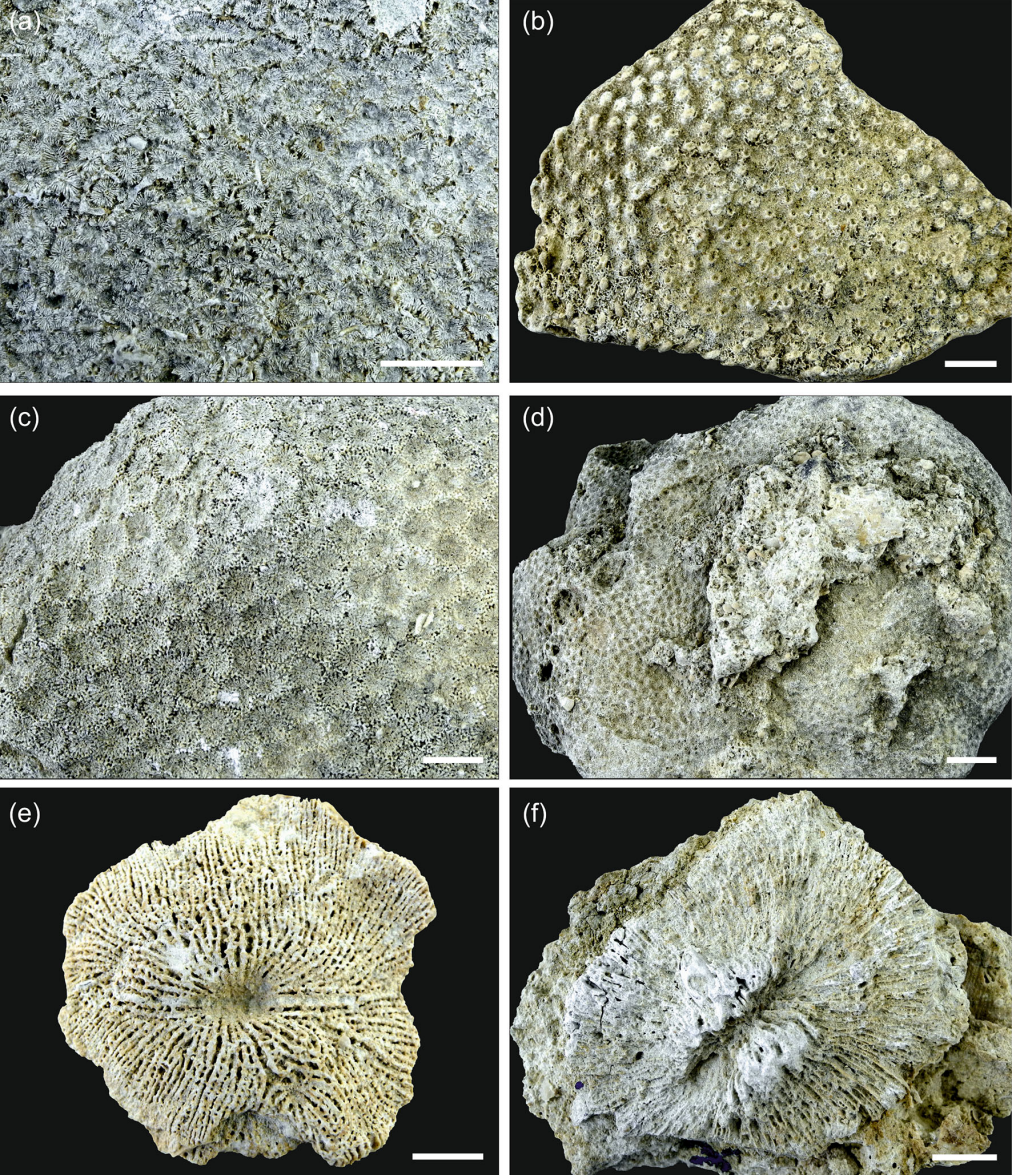
Representative corals from the studied assemblage. **a***Goniastrea edwardsi*. **b***Turbinaria mesenterina*. **c***Goniopora planulata*. **d***Porites* sp. **e***Lithophyllon* sp. **f** Fungiidae indet. The scale bar is always 1 cm

The majority of the corals at Mtwara are preserved in growth position indicating autochthonous deposition and fully marine conditions. A considerable open-marine, pelagic influence is also obvious from planktic foraminifers and calcareous nannoplankton in the muddy matrix between the corals. The high content of siliciclastic mud along with the dominance of coral taxa known to be well adapted to the physical and trophic characteristics of turbid coastal waters (*Fungia*, *Goniopora*, *Goniastrea*, *Oulophyllia*, *Pachyseris*, *Platygyra*, *Porites*, *Turbinaria*; Figs. 4, 5) (e.g. Stafford-Smith and Ormond 1992; Stafford-Smith 1993; Riegl et al. 1996; Tomascik et al. 1997; Wilson et al. 2005; Sofonia and Anthony 2008; Browne et al. 2012; Bessell-Browne et al. 2017; Johnson et al. 2017) points to a significant terrigenous influx. Furthermore, many coral colonies in the studied outcrop show growth features related to high, episodic sediment accumulation. Tabular-massive corals are characterized by ragged margins (Fig. 3b, c) that document events of sediment coverage and mortality in the marginal portion of the colony followed by phases of recovery and lateral colony expansion (Sanders and Baron-Szabo 2005). Other typical shapes of colonies grown under high episodic sedimentation include interconnected platy to laminar growth forms (Fig. 3f) and flat forms with scattered, protruding knobs (Fig. 3d, e; Sanders and Baron-Szabo 2005). Corals in nearshore turbid reefs also have to cope with low light conditions similar to mesophotic deep-water coral communities. For this reason, turbid reefs have a depth window of only a few metres (Morgan et al. 2016). Platy growth forms of corals, which dominate the sheetstone facies and are also abundant in the mix-platestone facies, are able to maximize light interception efficiency in poorly illuminated waters (Rosen et al. 2002; Sanders and Baron-Szabo 2005). Temporal changes in the local turbidity level are recorded by vertical alternations of sheetstone and mix-platestone facies (Figs. 2, 3a). The sheetstones are inferred to have formed under conditions of increased fine-gained siliciclastic sedimentation and reduced illumination. With decrease in terrestrial sediment supply, tabular-massive corals dominated the environment. The lack of rigid coral frameworks and distinct lateral ecological zonation patterns together with the near-horizontal decimetre- to metre-scale internal bedding (Figs. 2, 3a) indicate that the Mtwara bioconstruction represents a succession of low-relief biostromes that were each at least a few tens of metres in lateral extent and had risen less than a metre above the sea floor. Similar low-relief coral buildups are described from shallow (≤ 10 m), turbid-water settings at the delta front of the Miocene Mahakam Delta (East Kalimantan, Indonesia; Wilson and Lokier 2002; Wilson 2005; Novak et al. 2013; Santodomingo et al. 2015b).

### Palaeobiogeography

#### Biogeographic affinity

**Fig. 6:**
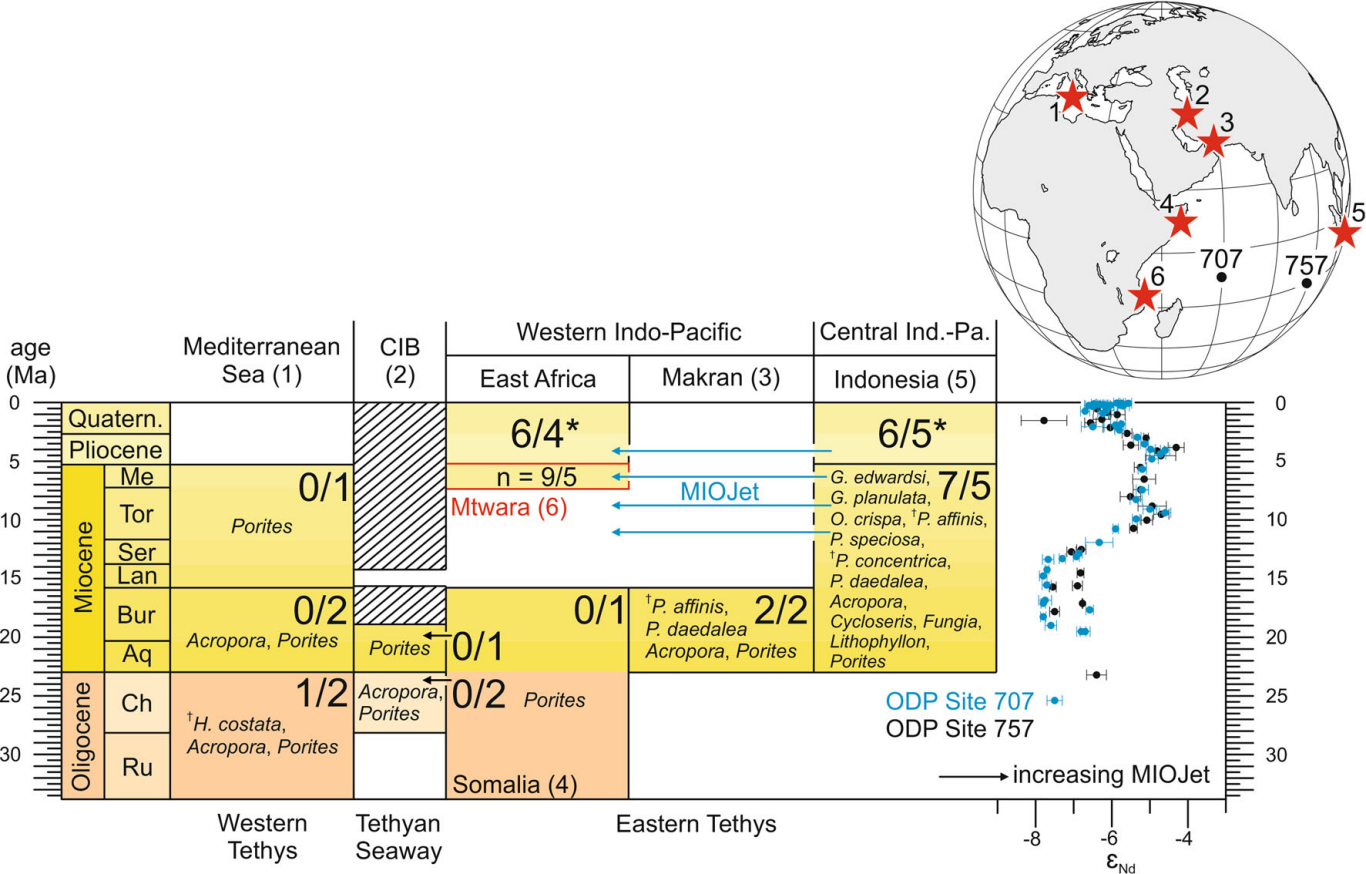
Summary chart comparing the Mtwara coral assemblage with Oligocene–Miocene and Recent coral faunas from the Mediterranean Sea, Tethyan Seaway (CIB = Central Iranian basins), and the Western and Central Indo-Pacific regions (*recent faunas). A total of nine species (^†^extinct species) and five genera of corals are identified at Mtwara locality (*n* = 9/5). The other numbers show the similarity of coral faunas from different geographic areas and stratigraphic units with Mtwara (species/genera which are common between both faunas according to Table 1). The hatching pattern shows the presence of landbridges between Africa–Arabia and Eurasia (based on Harzhauser et al. 2007). ε_Nd_ seawater records at ODP Sites 707 and 757 in the Indian Ocean indicate a westerly oceanic surface current (MIOJet) linking the eastern and western Indian Ocean from 14 to 3 Ma (Gourlan et al. 2008). The sources for the increase in the Nd radiogenic signatures lie to the east of the sites either in the Pacific Ocean or in the Sunda volcanic arcs; chronostratigraphy according to Gradstein et al. (2012)

The Messinian coral assemblage is of typical Indo-West Pacific composition. Thirteen of the sixteen identified taxa are still found in the Central Indo-West Pacific today and, except for *Lithophyllon* (Fig. 5e), also currently occur along the Tanzanian coast (Table 1). Three coral species (*Hydnophyllia costata*, *Pachyseris affinis*, *Platygyra concentrica*; Fig. 4a, c, e) are extinct (Table 1). Out of this group, *Hydnophyllia costata* (Fig. 4e) points to some relation with the Mediterranean region because except from the fossil reef site presented herein, it is only known from the Oligocene of northern Italy (Budd and Bosellini 2016). During Oligocene and early Miocene times, the Tethys connected the Atlantic and Pacific Oceans. Hydrogeographically, this marine connection existed until the Africa-Eurasia collision induced the closure of the Tethyan Seaway (present-day Middle East area) about 19 Ma ago (Burdigalian; Harzhauser et al. 2007; Fig. 6). The fossil record of *H. costata* shows that the species had a wide distribution in the Tethyan biogeographic realm before the Mediterranean and Indo-West Pacific separated. The high compositional similarity at the species level between the Messinian coral assemblage from Mtwara and the living coral fauna in the surrounding area (Fig. 6) suggest that the community structure of reef corals remained largely unchanged at the southern Tanzanian coast since the late Miocene.

#### Miocene patterns of faunal connectivity

**Fig. 7:**
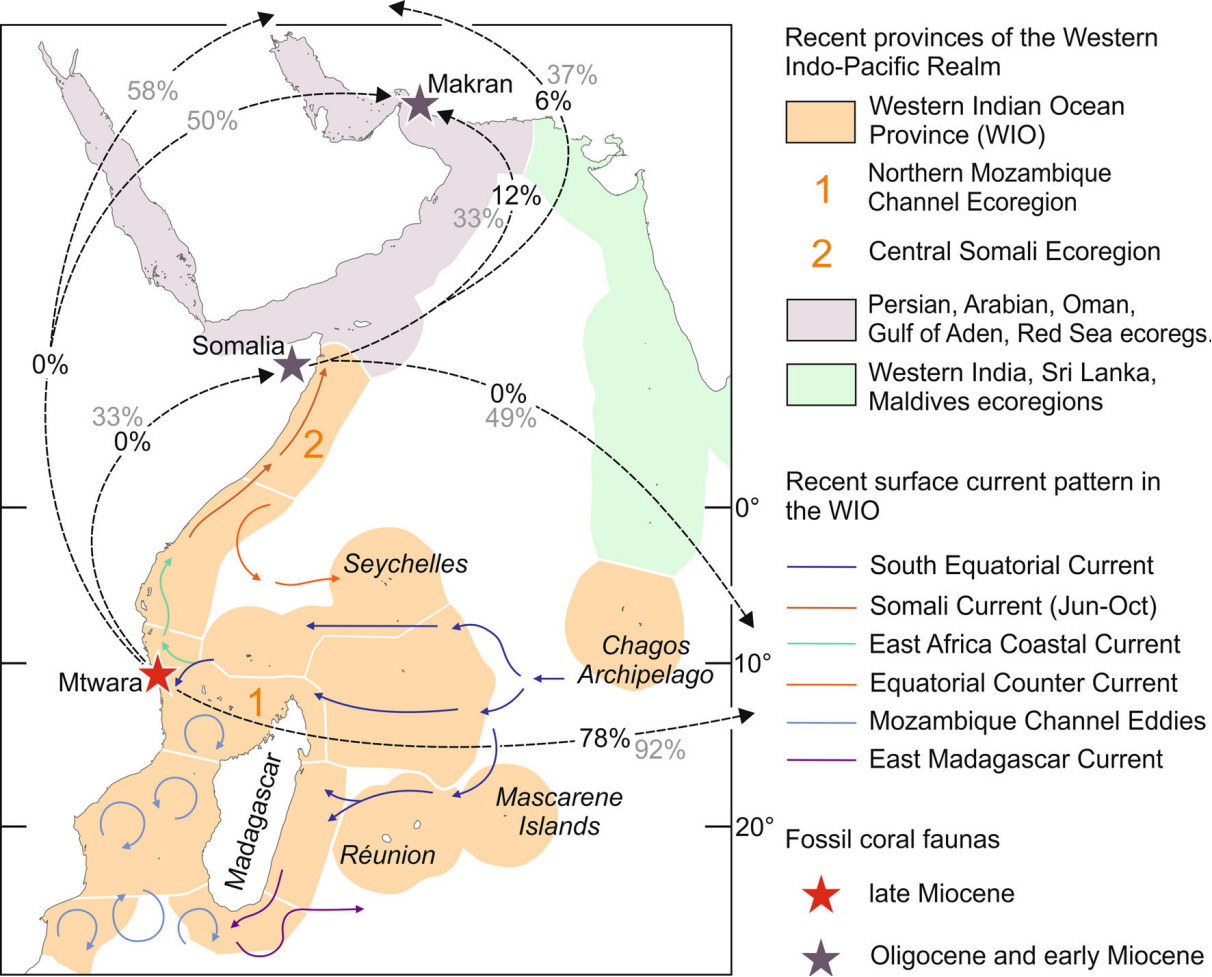
Boundaries and ecoregions (white lines) of the Recent Western Indian Ocean Province (WIO; orange area) based on species diversity and distribution of reef-building corals (according to Obura 2012). The principal surface currents in the WIO are indicated by coloured arrows and the black dashed arrows show the degree of faunistic overlap (black numbers = species level, grey numbers = genus level) between fossil coral faunas from different geographic regions and stratigraphic intervals (asterisks)

The Mtwara coral fauna shows a strong overlap (78%) at the species level, with faunas listed from turbid-water habitats in the Miocene of Indonesia, whereas faunistic relations with the Oligocene-early Miocene of Somalia (Latham 1929; Zuffardi-Comerci 1937; Azzaroli 1958; Bosellini et al. 1987) and the Central Iranian basins (Schuster and Wielandt 1999; Schuster 2002a, b; Yazidi et al. 2012) do not exist at the species level notwithstanding the closer spatial proximity (Figs. 6, 7; Table 1). The occurrences of *Pachyseris affinis* (Fig. 4a) and *Platygyra daedalea* (Fig. 4d) in the early Miocene of Makran (southeastern Iran; McCall et al. 1994) do not necessarily indicate a direct faunal connection with equatorial Eastern Africa because both species were also present in Indonesia during the late Miocene (Johnson et al. 2015; Fig. 6; Table 1).

The Oligocene-early Miocene coral record of Somalia (northeastern Africa), which is geographically closest to southern Tanzania (Figs. 6, 7), comprises 94 species of symbiotic reef corals (Supplementary Material 1). Although some high degree of taxonomic subjectivity may have biased the identification of fossil corals from Somalia, a faunistic relation with coeval faunas of Iran can be inferred (Fig. 7). Somalia has six symbiotic reef coral species in common with the Oligocene-early Miocene coral fauna of the Central Iranian basins (Schuster and Wielandt 1999; Schuster 2002a, b; Yazidi et al. 2012) and shares even 12 species with the early Miocene coral fauna of Makran (McCall et al. 1994; Supplementary Material 1). In contrast, the faunistic similarity between the Oligocene-early Miocene of Somalia and the Miocene of Indonesia is very low on the species level (2 species in common: *Hydnophora insignis*, *H. solidor*; Supplementary Material 1). The Indonesian record of *H. insignis* is Eocene (Bartonian) in age and that of *H. solidor* is Messinian or just Miocene in general (Johnson et al. 2015). Because of these stratigraphic offsets and their coeval occurrences in the Central Iranian basins (Schuster and Wielandt 1999) and Makran (McCall et al. 1994), the presence of *H. insignis* and *H. solidor* in Somalia provides evidence for a biogeographic connection with Iran rather than a faunistic link between Eastern Africa and the Malay Archipelago during the Oligocene-early Miocene. In accordance with our findings, Aquitanian and Burdigalian shallow-marine gastropod faunas from southern Tanzania, Oman, Makran and western India (Kutch and Kerala basins) show a strong provincialism in the Western Indo-West Pacific region and share only very little similarities with coeval faunas from the Central Indo-West Pacific at the species level (Harzhauser 2007, 2009, 2014; Harzhauser et al. 2009, 2017). At the genus level, the faunal relations are less pronounced, which may be due to the fact that the temporal and spatial ranges of taxonomic units are increasing with higher taxonomic rank, but a general affinity of the Mtwara coral fauna to Indonesia is still discernible (Fig. 7; Table 2; Supplementary Material 1).

**Table 2:**
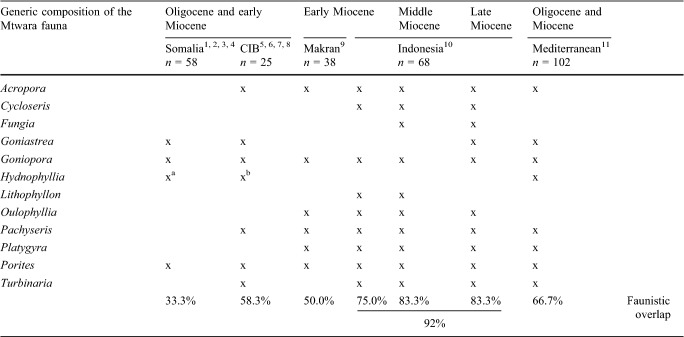
Faunistic affinity of the Mtwara coral assemblage with the Western (^1^Latham 1929; ^2^Zuffardi-Comerci 1937; ^3^Azzaroli 1958; ^4^Bosellini et al. 1987; ^5^Schuster and Wielandt 1999; ^6^Schuster 2002a, ^7^Schuster 2002b; ^8^Yazidi et al. 2012; ^9^McCall et al. 1994) and Central Indo-West Pacific (^10^Santodomingo et al. 2016) and Mediterranean (^11^Perrin and Bosellini 2012) regions at the genus level (*n* = number of symbiotic reef coral genera)

^a^*Hydnophyllia* was recorded by two species in the early Miocene of Somalia (*H. bellardii*, *H. intermedia*; Zuffardi-Comerci 1937). However, the plates in this monograph depict a *Variabilifavia ausuganensis* rather than a *H. bellardii* and the depicted *H. intermedia* looks more like a *H. sublabyrinthica* (see Budd and Bosellini 2016)

^b^Schuster (2002a) described two species of *Colpophyllia* (*C. longicollis*, *C*. *eocenica*) from the Qom Formation in central Iran that were synonymized with *Hydnophyllia scalaria* (Budd and Bosellini 2016)

The fossil coral faunas from Somalia and southern Tanzania have clear different biogeographic affinities (Fig. 7), which indicate an isolation of Eastern Africa from the Central Indo-West Pacific Region during the Oligocene-early Miocene and effective connectivity pathways for reef-building corals across the Indian Ocean during the Messinian. This points to a major biogeographic change in the Indian Ocean after the Burdigalian.

#### The process changing the biogeographic patterns

At present-day, the fossil reef sites in Tanzania and Somalia are located in different ecoregions of the Western Indian Ocean Province (Fig. 7). Based on species diversity and distribution of reef-building corals, the Western Indian Ocean Province is classified as the area including the East African coast between central Somalia and Delagoa Bay in Mozambique as well as Madagascar, the Seychelles, the Chagos Archipelago, the Mascarene Islands and the banks of the Mascarene Plateau (Obura 2012). Mtwara belongs to the Core Ecoregion of maximum coral richness in the northern Mozambique Channel region, and fossil localities in eastern Somalia (e.g. Bosellini et al. 1987) are part of the less diverse Central Somali Ecoregion. The reef coral faunas in the Persian, Arabian, Oman, Gulf of Aden and Red Sea ecoregions are differently composed and were grouped into a separate province (Obura 2012; Fig. 7). The Western Indian Ocean hotspot of coral diversity is maintained by the inflow of the South Equatorial Current, which brings coral larvae from the Malay Archipelago, and variable meso-scale eddies that confer a high coral reef connectivity within the area. Faunal export to the Central Somali ecoregion is enabled via the East Africa Coastal and Somali currents (Obura 2012; Fig. 7).

The biogeographic connection between Eastern Africa and Southeast Asia coincides with a major oceanographic reorganization during the middle Miocene. It resulted from the narrowing of the Indonesian Gateway, which initiated a large, strong, westward flowing surface and intermediate oceanic current, the Miocene Indian Ocean Equatorial Jet (MIOJet; Gourlan et al. 2008). Based on neodymium isotope evidence, it is suggested that this precursor of the present-day South Equatorial Current increased from 14 to 9 Ma, remained stable until 4 Ma and then decreased (Gourlan et al. 2008; Fig. 6).

The Fungiidae coral *Lithophyllon* is documented in the Central Indo-Pacific since the middle Miocene, but absent in the Western Indian Ocean (Table 1). Reports of *Lithophyllon* from the Burdigalian of Makran (Ghaedi et al. 2016: *L. floriformis persica*; Paleobiology Database, https://www.paleobiodb.org, accessed 09 April 2019: *Lithophyllon* sp., “*Lithophyllon*” *spinosa*) are doubtful. Both references refer to McCall et al. (1994) but there is no mention of the genus in this paper. Instead McCall et al. (1994) lists “*Lithophyllia*” *spinosa* and *Leptoseris* cf. *floriformis* from the concerned localities. *Leptoseris floriformis* is a synonym of *Lithophyllon undulatum* according to the Catalogue of Life (https://www.gbif.org, accessed 09 April 2019). The use of the abbreviation “cf.”, however, indicates that the specimen is in the genus *Leptoseris* and resembles *L. floriformis* but species identification cannot be certain. Therefore, it is likely that the early Miocene *Lithophyllon* records from Makran actually refer to *Lithophyllia* (a synonym of *Parascolymia*) and *Leptoseris*. The puzzling outpost of *Lithophyllon* in Tanzania (Fig. 5e) at ca 7–6 Ma might relate to the peak current strength of the MIOJet (Fig. 6). A stronger than present westward equatorial surface current between ca 9 and 4 Ma (Gourlan et al. 2008; Fig. 6) may have enhanced the long-term dispersal capability of *Lithophyllon* larvae by shortening their travel time. In the same way, the arrival of *Fungia*, which is not documented in the western Indian Ocean region for Oligocene and early Miocene times, in Eastern Africa (Tables 1, 2) was possibly also linked to the development of the MIOJet. However, *Fungia* remained living in the region until today unlike *Lithophyllon* (Fig. 6). The disappearance of *Lithophyllon* from the Western Indian Ocean Province after the Miocene might have been related to the Quaternary glacial–interglacial climate changes and associated reef coral range shifts and changing reef habitat availability and fragmentation (Kiessling et al. 2012; Pellissier et al. 2014; Lauchstedt et al. 2017). In particular, interglacial warming seems to have triggered substantial species range shifts away from the equator leading to a loss of equatorial reef coral diversity (Kiessling et al. 2012). The recolonization of the East African coast by *Lithophyllon* from refugia in the Central Indo-West Pacific during more favourable periods may have been precluded because of a weak South Equatorial Current compared to the MIOJet.

In conclusion, our results indicate that the Western Indian Ocean Province was not existent in its present form during the early Miocene and must have developed by the connection of the region to the Coral Triangle in Southeast Asia after the Burdigalian. This biogeographic change coincides with the onset and intensification of the Miocene Indian Ocean Equatorial Jet between 14 and 9 Ma in response to the narrowing of the Indonesian Gateway. The East African hotspot of coral diversity in the area of the northern Mozambique Channel thus formed during the middle to late Miocene as a satellite population of the Coral Triangle.

## Electronic supplementary material

Below is the link to the electronic supplementary material.

**Supplementary Material 1** List of symbiotic reef coral taxa described from the Oligocene and early Miocene of Somalia (NE Africa) and their occurrences in other geographic regions (x = species, G = genus) (XLSX 21 kb)

## References

[CR1] Azzaroli A (1958). L’Oligocene e il Miocene della Somalia. Palaeontographia Italica.

[CR2] Backman J, Raffi I, Rio D, Fornaciari E (2012). Biozonation and biochronology of Miocene through Pleistocene calcareous nannofossils from low and middle latitudes. Newsl Stratigr.

[CR3] Bessell-Browne P, Fisher R, Duckworth A, Jones R (2017). Mucous sheet production in *Porites*: an effective bioindicator of sediment related pressures. Ecol Indic.

[CR4] Bornhardt W (1900). Zur Oberflächengestaltung und Geologie Deutsch-Ostafrikas.

[CR5] Browne NK, Smithers SG, Perry CT (2012). Coral reefs of the turbid inner-shelf of the Great Barrier Reef, Australia: an environmental and geomorphic perspective on their occurrence, composition and growth. Earth Sci Rev.

[CR6] Bosellini A, Russo A, Arush MA, Cabdulqadir MM (1987). The Oligo – Miocene of Eil (NE Somalia): a prograding coral – *Lepidocyclina* system. J Afr Earth Sci.

[CR7] Budd AF, Bosellini FR (2016). Revision of Oligocene Mediterranean meandroid corals in the scleractinian families Mussidae, Merulinidae and Lobophyllidae. J Syst Palaeontol.

[CR8] Conesa GAR, Favre E, Münch P, Dalmasso H, Chaix C (2005). Biosedimentary and paleoenvironmental evolution of the Southern Marion Platform from the Middle to late Miocene (Northeast Australia, ODP Leg 194, Sites 1196 and 1199). ODP Sci Res.

[CR9] Duncan MP (1880). Sind fossils, corals and Alcyonaria. Paleontologia Indica.

[CR10] Förderer M, Rödder D, Langer MR (2018). Patterns of species richness and the center of biodiversity in modern Indo-Pacific larger foraminifera. Sci Rep.

[CR11] Ghaedi M, Johnson K, Yazdi M (2016). Paleoenvironmental conditions of Early Miocene corals, western Makran, Iran. Arab J Geosci.

[CR12] Gourlan AT, Meynadier L, Allègre CJ (2008). Tectonically driven changes in the Indian Ocean circulation over the last 25 Ma: Neodymium isotope evidence. Earth Planet Sci Lett.

[CR13] Gradstein FM, Ogg JG, Schmitz MD, Ogg GM (2012). The geologic time scale 2012.

[CR14] Gregory JW (1930) The fossil corals of Kenya colony collected by Miss McKinnon Wood. Monographs of the Geological Department of the Hunterian Museum, Glasgow University 4:185–209

[CR15] Harzhauser M (2007). Oligocene and Aquitanian gastropod faunas from the Sultanate of Oman and their biogeographic implications for the early western Indo-Pacific. Palaeontographica A.

[CR16] Harzhauser M (2009). Aquitanian gastropods of coastal Tanzania and their biogeographic implications for the early western Indo-Pacific. Palaeontographica A.

[CR17] Harzhauser M (2014). A seagrass-associated Early Miocene Indo-Pacific gastropod fauna from South West India (Kerala). Palaeontographica A.

[CR18] Harzhauser M, Reuter M, Mohtat T, Piller WE (2017). Early Miocene reef- and mudflat-associated gastropods from Makran (SE-Iran). Paläontol Z.

[CR19] Harzhauser M, Reuter M, Piller WE, Berning B, Kroh A, Mandic O (2009). Oligocene and early Miocene gastropods from Kutch (NW-India) document an early biogeographic switch from Western Tethys to Indo-Pacific. Paläontol Z.

[CR20] Harzhauser M, Kroh A, Mandic O, Piller WE, Göhlich U, Reuter M, Berning B (2007). Biogeographic responses to geodynamics: A key study all around the Oligo-Miocene Tethyan Seaway. Zool Anz.

[CR21] Hoegh-Guldberg O, Hoegh-Guldberg H, Veron JEN, Green A, Gomez ED, Lough J, King M, Ambariyanto Hansen L, Cinner J, Dews G, Russ G, Schuttenberg HZ, Peñaflor EL, Eakin CM, Christensen TRL, Abbey M, Areki F, Kosaka RA, Tewfik A, Oliver J (2009). The Coral Triangle and climate change: ecosystems, people and societies at risk.

[CR22] Hoeksema BW, Renema W (2007). Delineation of the Indo-Malayan centre of maximum marine biodiversity: the Coral Triangle. Biogeography, time and place: distributions, barriers, and islands.

[CR23] Insalaco E (1998). The descriptive nomenclature and classification of growth fabrics in fossil scleractinian reefs. Sediment Geol.

[CR24] Johnson KG, Hasibuan F, Müller W, Todd JA (2015). Biotic and environmental origins of the Southeast Asian marine biodiversity hotspot: the Throughflow Project. Palaios.

[CR25] Johnson JA, Perry CT, Smithers SG, Morgan KM, Santodomingo N, Johnson KG (2017). Palaeoecological records of coral community development on a turbid, nearshore reef complex: baselines for assessing ecological change. Coral Reefs.

[CR26] Kent PE, Hunt JA, Johnstone DW (1971) The geology and geophysics of coastal Tanzania. Institute of Geological Sciences Geophysical Paper 6, HMSO, London

[CR27] Key RM, Smith RA, Smelror RM, Sæther OM, Thorsnes T, Powell JH, Njange F, Zandamela EB (2008). Revised lithostratigraphy of the Mesozoic-Cenozoic succession of the onshore Rovuma Basin, northern coastal Mozambique. S Afr J Geol.

[CR28] Kiessling W, Simpson C, Beck B, Mewis H, Pandolfi JM (2012). Equatorial decline of reef corals during the last Pleistocene interglacial. PNAS.

[CR29] Latham MH (1929). Jurassic and Kainozoic corals from Somaliland. Trans R Soc Edinb.

[CR30] Lauchstedt A, Pandolfi JM, Kiessling W (2017). Towards a new paleotemperture proxy from reef coral occurrences. Sci Rep.

[CR31] Leprieur F, Descombes P, Gaboriau T, Cowman PF, Parravicini V, Kulbicki M, Melián CJ, de Santana CN, Heine C, Mouillot D, Bellood DR, Pellissier L (2016). Plate tectonics drive tropical reef biodiversity dynamics. Nat Commun.

[CR32] Mahajane ES (2014). The Davie Fracture Zone and adjacent basins in the offshore Mozambique Margin – a new insights for the hydrocarbon potential. Mar Pet Geol.

[CR33] Mahajane ES, Franke D (2014). The Rovuma Delta deep-water fold-and-thrust belt, offshore Mozambique. Tectonophysics.

[CR34] Martini E (1971) Standard Tertiary and Quaternary calcareous nannoplankton zonation. In: Farinacci A. (ed) Proceedings of the 2nd International Conference on Planktonic Microfossils 2, 739–785

[CR35] McCall J, Rosen B, Darell J (1994). Carbonate deposition in accretionary prism settings: Early Miocene coral limestones and corals of the Makran Mountain Range in southern Iran. Facies.

[CR36] Morgan KM, Perry CT, Smithers SG, Johnson JA, Daniell JJ (2016). Evidence of extensive reef development and high coral cover in nearshore environments: implications for understanding coral adaption in turbid settings. Sci Rep.

[CR37] Nicholas CJ, Pearson PN, McMillan IK, Ditchfield PW, Singano JM (2007). Structural evolution of southern coastal Tanzania since the Jurassic. J Afr Earth Sci.

[CR38] Novak V, Santodomingo N, Rösler A, Di Martino E, Braga JC, Taylor PD, Johnson KG, Renema W (2013). Environmental reconstruction of a late Burdigalian (Miocene) patch reef in deltaic deposits (East Kalimantan, Indonesia). Palaeogeogr Palaeoclimatol Palaeoecol.

[CR39] Obura D (2012). The diversity and biogeography of Western Indian Ocean reef-building corals. PLoS One.

[CR40] Okada H, Bukry D (1980). Supplementary modification and introduction of code numbers to the low-latitude coccolith biostratigraphic zonation (Bukry 1973, 1975). Mar Micropaleontol.

[CR41] Pellissier L, Leprieur F, Parravicini V, Cowman PF, Kulbicki M, Litsios G, Olsen SM, Wisz MS, Bellwood DR, Mouillot D (2014). Quaternary coral reef refugia preserved fish diversity. Science.

[CR42] Perch-Nielsen K, Bolli HM, Saunders JB, Perch-Nielsen K (1985). Cenozoic calcareous nannofossils. Plankton stratigraphy.

[CR43] Perrin C, Bosellini FR (2012). Paleobiogeography of scleractinian reef corals: changing patterns during the Oligocene-Miocene climatic transition in the Mediterranean. Earth Sci Rev.

[CR44] Reaka M, Rodgers P, Kudla AU (2008). Patterns of biodiversity and endemism on Indo-West Pacific coral reefs. PNAS.

[CR45] Renema W, Bellwood DR, Braga JC, Bromfield K, Hall R, Johnson KG, Lunt P, Meyer CP, McMonagle LB, Morley RJ, O’Dea A, Todd JA, Wesselingh FP, Wilson MEJ, Pandolfi JM (2008). Hopping hotspots: global shifts in marine biodiversity. Science.

[CR46] Riegl B, Heine C, Branch GM (1996). Function of funnel-shaped coral growth in a high-sedimentation environment. Mar Ecol Prog Ser.

[CR47] Roberts EM, Stevens NJ, O’Connor PM, Dirks PHGM, Gottfried MD, Clyde WC, Armstrong RA, Kemp AIS, Hemming S (2012). Initiation of the western branch of the East African Rift coeval with the eastern branch. Nat Geosci.

[CR48] Rosen BR, Aillud GS, Bosellini FR, Clack NJ, Insalaco E (2002) Platy coral assemblages: 200 million years of functional stability in response to the limiting effects of light and turbidity. Proc 8th Int Coral Reef Symp 1:255–264

[CR49] Salman G, Abdula I (1995). Development of the Mozambique and Ruvuma sedimentary basins, offshore Mozambique. Sediment Geol.

[CR50] Sanders D, Baron-Szabo RC (2005). Scleractinian assemblages under sediment input: their characteristics and relation to the nutrient input concept. Palaeogeogr Palaeoclimatol Palaeoecol.

[CR77] Santodomingo N (2014) Miocene reef-coral diversity of Indonesia: unlocking the murky origins of the Coral Triangle. Ph.D. thesis, Utrecht University, p 340

[CR51] Santodomingo N, Wallace CC, Johnson KG (2015). Fossils reveal a high diversity of the staghorn coral genera *Acropora* and *Isopora* (Scleractinia: Acroporidae) in the Neogene of Indonesia. Zool J Linn Soc.

[CR52] Santodomingo N, Novak V, Pretković V, Marshall N, Di Martino E, Lo Giudice Capelli E, Rösler A, Reich S, Braga JC, Renema W, Johnson KG (2015). A diverse patch reef from turbid habitats in the middle Miocene (East Kalimantan, Indonesia). Palaios.

[CR53] Santodomingo N, Renema W, Johnson KG (2016). Understanding the murky history of the Coral Triangle: Miocene corals and reef habitats in East Kalimantan (Indonesia). Coral Reefs.

[CR54] Schlüter T (1997). Geology of East Africa.

[CR55] Schuster F, Wielandt U (1999). Oligocene and Early Miocene coral faunas from Iran: palaeoecology and palaeobiogeography. Int J Earth Sci.

[CR56] Schuster F (2002). Scleractinian corals from the Oligocene of the Qom Formation (Esfahan-Sirjan fore-arc basin, Iran. Cour Forsch-Inst Senckenberg.

[CR57] Schuster F (2002). Early Miocene scleractinian corals from Qom and Asmari formations (central and southwest Iran. Cour Forsch-Inst Senckenberg.

[CR58] Smelror M, Key R, Daudi E, Njange F (2006). Frontier with high expectations. GeoExpro.

[CR59] Smelror M, Key RM, Smith RA, Njange F (2008). Late Jurassic and Cretaceous palynostratigraphy of the onshore Rovuma Basin, northern Mozambique. Palynology.

[CR60] Sofonia JJ, Anthony KRN (2008). High-sediment tolerance in the reef coral *Turbinaria mesenterina* from the inner Great Barrier Reef lagoon (Australia). Estuar Coast Shelf Sci.

[CR61] Spalding MD, Fox HE, Allen GR, Davidson N, Ferdaña ZA, Finlayson M, Halpern BS, Jorge MA, Lombana A, Lourie SA, Martin KD, McManus E, Molnar J, Recchia CA, Robertson J (2007). Marine ecoregions of the world: a bioregionalization of coastal and shelf areas. BioScience.

[CR62] Stafford-Smith MG (1993). Sediment-rejection efficiency of 22 species of Australian scleractinian corals. Mar Biol.

[CR63] Stafford-Smith MG, Ormond RFG (1992). Sediment-rejection mechanisms of 42 species of Australian scleractinian corals. Australian Journal of Marine and Freshwater Research.

[CR64] Tittensor DP, Mora C, Jetz W, Lotze HK, Ricard D, Vanden Berghe E, Worm B (2010). Global patterns and predictions of marine biodiversity across taxa. Nature.

[CR65] Tomascik T, Mah AJ, Nontji A, Moosa MK (1997). The ecology of Indonesian seas, part 2.

[CR66] Veron JEN (2000). Corals of the world.

[CR67] Veron JEN, Stafford-Smith M, DeVantier L, Turak E (2015). Overview of distribution patterns of zooxanthellate Scleractinia. Front Mar Sci.

[CR68] Wade BS, Pearson PN, Berggren WA, Pälike H (2011). Review and revision of Cenozoic tropical planktonic foraminiferal biostratigraphy and calibration to the geomagnetic polarity and astronomical time scale. Earth Sci Rev.

[CR69] Wallace CC, Bosellini FR (2014). Acropora (Scleractinia) from the Oligocene and Miocene of Europe: species longevity, origination and turnover following the Eocene-Oligocene transition. J Syst Palaeontol.

[CR70] Wielandt-Schuster U, Schuster F, Harzhauser M, Mandic O, Kroh A, Rögl F, Reisinger J, Liebetrau V, Steininger FF, Piller WE (2004). Stratigraphy and palaeoecology of Oligocene and Early Miocene sedimentary sequences of the Mesohellenic Basin (NW Greece). Cour Forsch-Inst Senckenberg.

[CR71] Wilson MEJ (2005). Development of equatorial delta-front patch reefs during the Neogene, Borneo. J Sediment Res.

[CR72] Wilson MEJ, Rosen BR, Hall R, Holloway JD (1998). Implications of paucity of corals in the Paleogene of SE Asia: plate tectonics or Centre of Origin?. Biogeography and geological evolution of SE Asia.

[CR73] Wilson MEJ, Lokier SW (2002). Siliciclastic and volcaniclastic influences on equatorial carbonates: insights from the Neogene of Indonesia. Sedimentology.

[CR74] Wilson JJ, Marimuthu N, Kumaraguru AK (2005). Sedimentation of silt in the coral reef environment of Palk Bay, India. J Mar Biol Assoc India.

[CR75] Yazidi M, Shirrazi MP, Rahiminejad AH, Motavalipoor R (2012). Paleobathymetry and paleoecology of colonial corals from the Oligocene-early Miocene (?) Qom Formation (Dizlu area, central Iran). Carbonate Evaporite.

[CR76] Zuffardi-Comerci R (1937). Corallari oligocenici e miocenici della Somalia. Palaeontographia Italica.

